# A quest for long-distance signals: the epidermis as central regulator of pipecolic acid-associated systemic acquired resistance

**DOI:** 10.1093/jxb/eraa606

**Published:** 2021-03-29

**Authors:** A Corina Vlot

**Affiliations:** Helmholtz Zentrum München, Institute of Biochemical Plant Pathology, Ingolstaedter Landstr. 1, D-85764 Neuherberg, Germany

**Keywords:** ALD1, pipecolic acid, plant immunity, systemic acquired resistance

## Abstract

This article comments on:

**Jiang SC, Engle NL, Banday ZZ, Cecchini NM, Jung HW, Tschaplinski TJ, Greenberg JT**. 2021. ALD1 accumulation in Arabidopsis epidermal plastids confers local and non-autonomous disease resistance. Journal of Experimental Botany 72, 2710–2726.

**AGD2-like DEFENSE RESPONSE PROTEIN 1 (ALD1) is an aminotransferase that is necessary for the biosynthesis of the immune-active non-protein amino acid pipecolic acid (Pip). Pip and its *N*-hydroxylated derivative, *N*-hydroxy-Pip (NHP), have been suggested as possible long-distance signals moving in plants from infected to systemic, uninfected sites to enhance immunity**. Jiang *et al.* (2021)**show that accumulation of ALD1 in epidermal chloroplasts at local, infected sites promotes systemic immunity. Their results highlight the epidermis as a site of active immune signaling and ALD1 as an important upstream regulator of long-distance signal transmission in systemic acquired resistance (SAR).**

The establishment of SAR, an induced state of defense in the systemic, healthy tissues of locally infected plants, depends on the defense-associated phytohormone salicylic acid (SA) and its presumably synergistic interaction with a parallel pathway driven by Pip/NHP (Vlot *et al.*, 2020). Since it became clear >25 years ago that SA accumulation in systemic tissues is more important for SAR than SA’s systemic translocation, the scientific quest for the identity of other or additional long-distance signal(s) of SAR has been ongoing (Vernooij *et al.*, 1994; Lim *et al.*, 2020; Vlot *et al.*, 2020). Pip was originally identified in petiole exudates of SAR-induced plants, and is systemically mobile (Navarova et al., 2012; C. Wang *et al.*, 2018). For this reason, Pip and, more recently, its derivatives NHP, which also moves systemically in plants, and/or NHP-glycoside are among the ‘hottest’ candidates to function as long-distance signals of SAR (see Box 1) (Navarova et al., 2012; Chen *et al.*, 2018; Hartmann *et al.*, 2018).

**Box 1. The role of ALD1 in systemic acquired resistance (SAR)**

SAR is a form of induced resistance that is activated in systemic, healthy tissues of plants undergoing a localized infection (Vlot *et al.*, 2020). During the establishment of SAR, both salicylic acid (SA) and pipecolic acid (Pip) accumulate in local, infected, and systemic tissues (Vlot *et al.*, 2009; Navarova et al., 2012). The SA and Pip pathways perform parallel and synergistic functions in SAR (Vlot *et al.*, 2020). Pip is synthesized from l-lysine by two consecutive enzymatic steps catalyzed by the aminotransferase ALD1 and the reducing factor SAR-DEFICIENT4 (SARD4) in the chloroplast (Ding *et al.*, 2016; Hartmann *et al.*, 2017). Subsequent *N*-hydroxylation by FLAVIN-DEPENDENT MONOOXYGENASE1 (FMO1) in the cytosol results in the accumulation of *N*-hydroxy-Pip (NHP) (Chen *et al.*, 2018; Hartmann *et al.*, 2018). Because *fmo1* mutant plants do not mount an induced resistance response to exogenously applied Pip, FMO1-derived NHP is believed to act as the bioactive derivative of Pip in defense (Chen *et al.*, 2018; Hartmann *et al.*, 2018). *ald1* mutant plants are affected in Pip biosynthesis, and consequently accumulate neither Pip nor NHP or its glycosylated derivative. Pip, and presumably NHP, drives a SAR signaling pathway that results in the release of other potentially mobile SAR signals, including azelaic acid (AzA) and glycerol-3-phosphate (G3P) (C. Wang *et al.*, 2018). Pip and G3P further cooperate upstream of the emission of SAR-inducing volatile organic compounds (VOCs) from infected tissues. Jiang *et al.* (2021) show that ALD1 accumulation, and thus consequently Pip/NHP, in epidermal chloroplasts influences ‘local’ or autonomous disease resistance as an immediate response to infection in both local and systemic tissues. Additionally, local ALD1 acts upstream of the release of a long-distance signal of SAR. Ongoing and future research will tell if this long-distance SAR signal includes Pip, NHP, and/or other signaling molecules, such as SA, AzA, G3P, or volatile SAR cues.

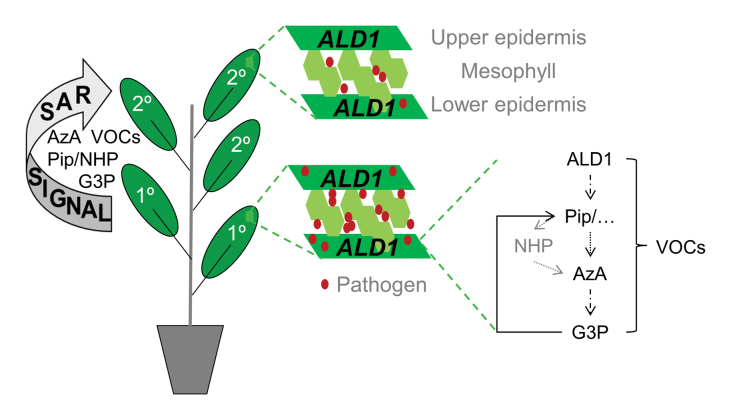

Jiang *et al.* (2021) exploited the conditional expression of an *ALD1* transgene from a dexamethasone (DEX)-inducible promoter to study the role of ALD1 in disease resistance and SAR. The authors found that expression of *ALD1* at the site of a local infection complemented the local/basal disease resistance phenotype caused by the *ald1* mutation. Further, upon a local infection, accumulation of ALD1 at the infection site sufficed to enhance the immune status of systemic tissues, a status Jiang *et al.* (2021) termed the ‘response gain of SAR’. Importantly, these data support the hypothesis that ALD1 acts upstream of SAR signal generation.

The response gain of SAR derives from a mathematical model, which allows Jiang *et al.* (2021) to differentiate between spatially distinct, local, and systemic roles of ALD1 in SAR. In doing so, this is the first study to show that ALD1, and thus consequently Pip/NHP, indeed promotes systemic immunity in a two-step incremental process. First, ALD1 acts in local immunity and essentially regulates the release of one or more long-distance SAR signals. Second, after the arrival of this/these signals in the systemic tissue, subsequent immune responses are induced. This response gain of SAR is observed in the absence of ALD1. Finally, however, full defense is only induced in the presence of ALD1, presumably through the influence of ALD1 on local or immediate defenses, which are triggered in response to the secondary inoculum.

Many studies exploit petiole exudates of SAR-induced plants to study the potential importance of SAR-associated genes in local, infected compared with systemic tissues (reviewed in Vlot *et al.*, 2020). In such approaches, petiole exudates from infected *ald1* mutant plants were shown to contain signals that confer SAR to wild-type recipient plants, whereas *ald1* mutant plants did not mount a normal SAR response to petiole exudates from infected wild-type plants (C. Wang *et al.*, 2018). These findings argue in favor of a role for ALD1 and Pip in systemic SAR signal perception or propagation, and against a role for Pip or its derivatives as long-distance signals. In contrast, a local induction of *ALD1-*dependent Pip accumulation downstream of mitogen-activated protein kinase kinase 4 is sufficient to confer SAR, supporting a possible role for Pip in SAR signal generation (Y. Wang *et al.*, 2018). In line with this hypothesis, the Greenberg group showed in earlier work that ubiquitous overexpression of *ALD1* leads to the accumulation of SAR-inducing signals in petiole exudates in the absence of infection, suggesting that ALD1 alone suffices to trigger SAR signal generation (Cecchini *et al.*, 2015a). Notably though, Pip could not be detected in these petiole exudates, and the authors proposed that ALD1 is involved in the biosynthesis of additional non-Pip metabolites that are important for SAR (Cecchini *et al.*, 2015a). Indeed, Hartmann *et al.* (2017) showed that ALD1-associated reaction products other than Pip are possible. Similarly, Jiang *et al.* (2021) could not detect Pip or NHP in petiole exudates of leaves accumulating ALD1 in epidermal cells. It thus seems possible that ALD1-associated non-Pip metabolites contribute to the response gain of SAR.

Notably, it cannot be excluded that Pip and/or NHP simply did not accumulate to detectable levels in petiole exudates of *ALD1*-expressing leaves in the absence of infection (Cecchini *et al.*, 2015a) or in infected leaves accumulating ALD1 in epidermal cells only (Jiang *et al.*, 2021). It is not impossible that such low levels of Pip/NHP or the accumulation of other ALD1-associated metabolites were sufficient to act as long-distance signals to drive SAR. Alternatively, Pip acts upstream in SAR cascades driving the accumulation of other potentially phloem-mobile SAR signals, including azelaic acid and glycerol-3-phosphate (Jung *et al.*, 2009; Chanda *et al.*, 2011; C. Wang *et al.*, 2018). In addition, Pip cooperates with glycerol-3-phosphate to promote the emission of volatile organic compounds from infected tissues (Wenig *et al.*, 2019). These airborne molecules are recognized as SAR cues in systemic tissues of the same and distal plants, and result in a response gain of SAR in the absence of ALD1 (Wenig *et al.*, 2019). Thus, the hypothesis should remain under consideration that Pip/NHP drive SAR signal generation or transmission, but are not themselves or alone long-distance signals of SAR (see Box 1).

*Pseudomonas syringae* propagates in association with epidermal cells, but more prominently in the intercellular space of the mesophyll. Therefore, it is striking that accumulation of ALD1 in the chloroplasts of epidermal cells suffices for a full complementation of the defense- and SAR-compromised phenotypes of *ald1* mutant plants (Jiang *et al.*, 2021). Consequently, ALD1 and thus Pip/NHP or other ALD1-derived metabolites might contribute to cell-to-cell-based local and systemic signaling mechanisms. In doing so, ALD1 might cooperate with other defense-associated factors that accumulate in epidermal cells and are, for example, involved in SA biosynthesis (Seguel *et al.*, 2018) and SAR (Cecchini *et al.*, 2015b). These might act together in a process termed by Jiang *et al.* (2021) as ‘skin-mediated systemic defense’. Possibly, ALD1 mitigates responses in the epidermis forwarding cell-to-cell cues to and/or from the mesophyll and, through its role in the accumulation of Pip, NHP, or other unknown metabolites, also to systemic tissues. The epidermal cell layer is in a prime position to connect to the cuticle, which was recently proposed to function as an important conduit of long-distance signals in SAR (Lim *et al.*, 2020). Perhaps a future focus on SAR-associated responses in epidermal cells will fast-forward the quest for the long-distance SAR signal, and answer the question of whether this signal is an as yet unknown metabolite or includes one or more of the known suspects Pip, NHP, azelaic acid, glycerol-3-phosphate, volatile organic compounds, SA, and more!
